# Supplementary material for: Morphology and structure of ZIF-8 during crystallisation measured by dynamic angle-resolved second harmonic scattering
Source: Nat Commun. 2018 Aug 24;9:3418. doi: 10.1038/s41467-018-05713-4 (PMC6109061; doi:10.1038/s41467-018-05713-4)
Supplement: Supplementary file 1 — Supplementary Information [file 41467_2018_5713_MOESM1_ESM.pdf]

Supporting information

Morphology and structure of ZIF-8 during crystallisation measured by dynamic angle-resolved second harmonic scattering

Stijn Van Cleuvenbergen *et al.*

Supplementary Note 1. Alignment procedure and data treatment

The collection system was aligned with low intensity 532 nm laser light that had been spatially filtered to produce a Gaussian beam profile. The intensity was controlled by polarization optics. The alignment procedure consists of firstly bringing the laser beam in the middle (0,0) of the chip of the EM-CCD camera, then focusing L3 (see Figure 1) on infinity by bringing the collimated laser beam into focus, the position is adjusted so the focal point coincides with (0,0). L2 and L1 are aligned on axis subsequently by insertion in the optical path and adjusting until the center of the image coincides with (0,0) as well, while the back reflections are used to ensure proper alignment. Then the distance between L1 and L2 is adjusted so that the transmitted laser light is in collimation, which can be verified by projection or by using a sheer plate. Finally the middle of the cuvette is placed at the focal point of L1, overlapping with the focal point of the input lens focusing the laser light.

To convert pixel position on the EM-CCD camera to scattering angle a 1D transmission grating (Thorlabs, 300 gvs/mm) was inserted at the position of the cuvette (Supplementary Figure 1a). We made use of an alignment laser to achieve different diffraction peaks on the camera chip. Using the grating equation the angles for the different diffraction peaks (for which the pixel position is known) were calculated and a linear extrapolation was performed to achieve pixel-to-angle conversion (Supplementary Figure 1b). Note here that a correction for the refractive index of the solvent is necessary when calibrating for scattering experiments, the diffraction angles are found as:

$$\theta_m = \mathrm{Arcsin}(\frac{m\lambda}{nd}) \quad (1)$$

With m the order, λ the wavelength, d the grating spacing and n the refractive index of the used solvent.

In our experiments we performed binning in the software rather than by image analysis, since this reduces the signal to noise ratio of the measurement. Typically we binned over an area of 4 by 15 pixels, which agrees with an angular resolution of about 0.7° and an out of plane collection angle of about 2.6°.

Dark correction was performed by measuring for an open beam without sample present.

The data are finally corrected for (divided by) a function accounting for the difference in collection efficiency at different angles, since at higher angles there tends to be a drop-off. In order to achieve this the hyper-Rayleigh signal of the bare solvent is gathered, and fitted with a high-order polynomial which is used as a calibration function. (Supplementary Figure 1c)

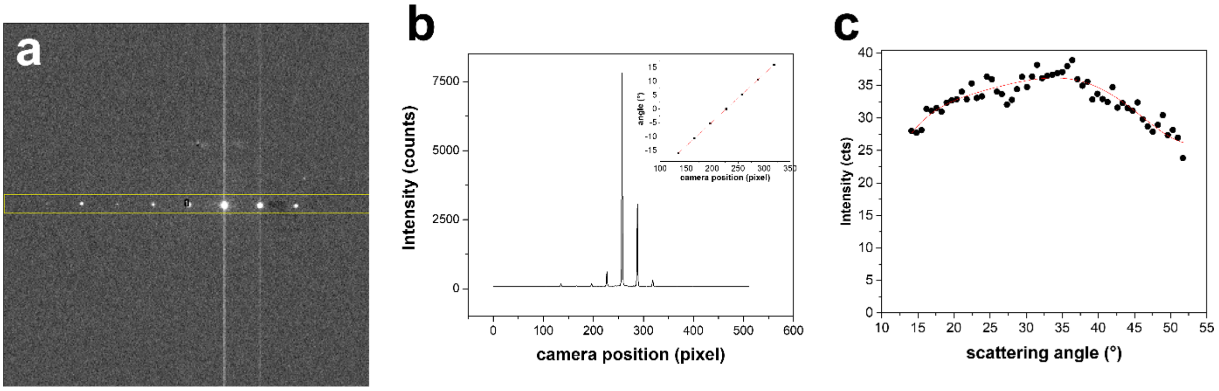

Supplementary Figure 1 Calibration of angle and collection efficiency.. a) Image of a 300 gvs/mm grating at normal incidence for 410 nm, the image is 512 pixels wide, b) Graph of selected area (yellow) in image 2a. The inset shows a linear extrapolation (red line) to achieve pixel-to-angle conversion. The values here are corrected for the refractive index of water. In our experiments the angles are then adjusted afterwards for the non-zero angle of incidence (e.g. 34° here). c) Hyper-Rayleigh scattering from methanol in order to calibrate the collection system. The data were fitted to a fifth-order polynomial ($y=a_0+a_1x+a_2x^2+ a_3x^3+ a_4x^4+ a_5x^5$).

Supplementary Note 2. AR-SHS resulting from the surface of polystyrene beads coated with Malachite green

To validate the performance of the AR-SHS setup, we measured a series of polystyrene beads of known sizes coated with malachite green dye molecules. Once the positively charged dye is adsorbed, aided by the negatively charged sulfate groups at the surface of the polystyrene beads, coherent effects between malachite green molecules result in a strong AR-SHS signal. The coherent AR-SHS signal of coated polystyrene beads is at least 2 orders of magnitude greater than the incoherent second harmonic scattering signal (i.e. hyper-Rayleigh scattering) of solutions of malachite green of the same concentration, depending on the size of the beads.[1] We measured polystyrene beads of 170, 320 and 490 nm in size, the resulting patterns are shown in Supplementary Figure 2. As expected for AR-SHS patterns from the surface of spheres, the intensity drops off towards low scattering angles to become zero in the forward direction. The larger the spheres, the more the position of the first maximum in the scattering pattern shifts towards lower angles. We used the Rayleigh-Gans-Debye model developed by Yang *et al.* to validate our data.[2] In this model the field inside the particle is set equal to that of the input beam and malachite green molecules are assumed to point radially outward from the surface and have a dominant radial component along their molecular radial axis. While more intricate models have been developed, based on nonlinear Mie theory or taking into account the full tensorial nature of the first hyperpolarizability of malachite green, this simple model predicts AR-SHS patterns well as long as the size of the spheres is relatively small compared to the wavelength, as can be verified in the aforementioned paper.[2] The scattering patterns for the 3 different sizes of malachite green coated polystyrene beads are indeed in good agreement with the theoretically expected curves (red line Supplementary Figure 2). To generate these curves the size was fixed at the known size of the polystyrene beads, and the wavelength set to 1030 nm, a scaling factor was then used to match the data.

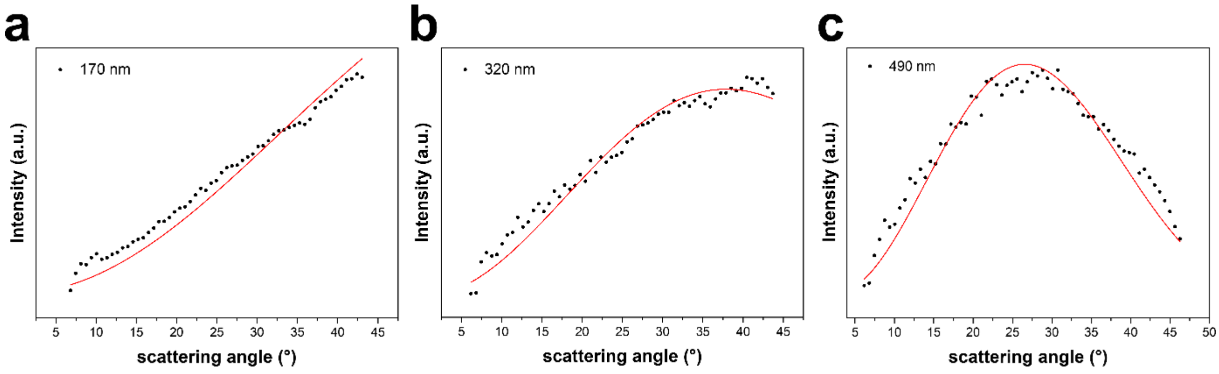

Supplementary Figure 2. AR-SHS patterns from the surface of malachite green coated polystyrene beads of different sizes.The diameters are a) 170 nm, b) 320 nm and c) 490 nm.The red lines represent the theoretically expected pattern according to the model of Yang *et al.*[2] Integration time is 3 seconds. The selected polarization combination is PSS.

Supplementary Note 3. Nonlinear Rayleigh-Gans-Debye model for AR-SHS resulting from the bulk of crystalline domains

The model adapted from de Beer *et al.* (reference 34) describes the nonlinear response for 1 crystalline domain at the detector (located at r_0) as:

$$\varepsilon^{(2)}_{u_0u_1u_2}(r_0) \propto \varepsilon_{u_1}\varepsilon_{u_2} e^{ik_{2\omega}r_0} \frac{k_0^2}{r_0} F(qR)G(\theta;\psi,\xi,\zeta) \quad (2)$$

$$F(qR) = \int_V e^{i\mathbf{q}.\mathbf{r}'} d^3\mathbf{r}' = 4\pi R^3 \frac{\sin(qR) - qR\cos(qR)}{(qR)^3} \quad (3)$$

$$G(\theta;\psi,\xi,\zeta) = \sum_{\alpha_0,\alpha_1,\alpha_2} \chi^{(2)}_{\alpha_0,\alpha_1,\alpha_2} \prod_{i=0}^{2} (\Re(\mathbf{e}'_{\alpha_j}).\mathbf{e}_{u_i}) \quad (4)$$

With ε the electric field amplitude, $\mathbf{e}_{u_i}$ the unit vector for polarization state u_i of incoming (i=1,2) and outgoing (i=0) light, k_0 the magnitude of the wavevector of the scattered SHS light, and **q** the scattering wave vector defined here as ($\mathbf{k}_0$-2$\mathbf{k}_\omega$). The magnitude of the scattering wave vector is then found as q=2$k_0$sin(Θ /2), with k_0 the magnitude of the wave vector of the scattered second harmonic light. The response can be split into a domain size dependent part (*F(qR)*) and a part that depends on the nonlinear optical properties (*G(Θ;ψ,ξ,ς)*). α_i represents the orthonormal base for crystalline axes *x*, *y* and *z*. $\Re$ represents a rotation over Euler angles *ψ*,*ξ* and *ς* describing the crystalline domain for an arbitrary orientation. *V* is the volume of the domain and is approximated as a sphere.

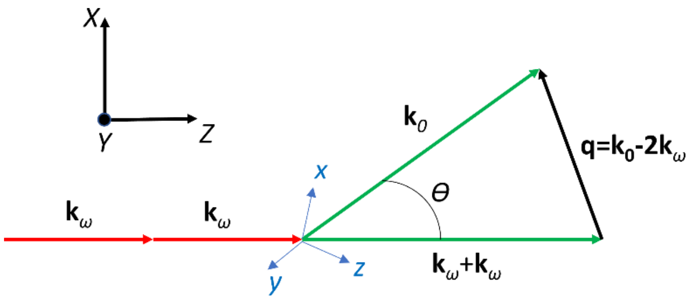

Supplementary Figure 3. Top view (horizontal scattering plane) of the AR-SHS experiment. The macroscopic axes are depicted *X*,*Y*,*Z*. In blue the local framework (*x*,*y*,*z*) is depicted for an individual crystallite adopting a random orientation. The wave vectors of the incident light fields ($\mathbf{k}_\omega$, degenerate), the scattered (detected) light ($\mathbf{k}_0$) and the sum of the incident light wave vectors (2$\mathbf{k}_\omega$) are depicted, as well as the scattering wave vector **q**.

In our experiments the incoming fields are degenerate so $\mathbf{e}_{u_1} = \mathbf{e}_{u_2}$. The unit vectors describing polarization state $\mathbf{u}_i$ are then found as:

$\mathbf{e}_{u_{0s}}$ =(0,1,0)= $\mathbf{e}_{u_{1s}}$ = $\mathbf{e}_{u_{2s}}$; $\mathbf{e}_{u_{1p}}$ = $\mathbf{e}_{u_{2p}}$ =(1,0,0); $\mathbf{e}_{u_{0p}}$ =(cos(Θ),0,sin(Θ)).

The projections $\Re(\mathbf{e}'_{\alpha_j})$ of the unit vectors of the local onto the macroscopic framework correspond to:

$$\Re(\boldsymbol{e}') = \begin{pmatrix} \Re_{Xx} & \Re_{Xy} & \Re_{Xz} \\ \Re_{Yx} & \Re_{Yy} & \Re_{Yz} \\ \Re_{Zx} & \Re_{Zy} & \Re_{Zz} \end{pmatrix} .\boldsymbol{e}' \quad (5)$$

With $\mathcal{R}_{Ij}$ the different elements of the Euler transformation matrix depending on Euler angles ψ,ξ,ς. $\mathcal{R}(\boldsymbol{e}_x')$then becomes $(\mathcal{R}_{Xx}, \mathcal{R}_{Xy}, \mathcal{R}_{Xz})$. Now we can evaluate the contribution for every tensor element of $\chi^{(2)}$ on G(Θ;ψ,ξ,ς). For instance for $\chi^{(2)}_{xyz}$ we find:

$$\chi^{(2)}_{xyz}(\Re(\mathbf{e}_x').\mathbf{e}_{u_0})(\Re(\mathbf{e}_y').\mathbf{e}_{u_1})(\Re(\mathbf{e}_z').\mathbf{e}_{u_2}) \quad (6)$$

G(Θ;ψ,ξ,ς) is then found as the sum over all contributions/ tensor elements.

The total scattered intensity for each polarization combination is then found as the incoherent sum over all N scatterers:

$$I_{u_0,u_1,u_2}(r_0) \propto N\varepsilon_{u_1}^2\varepsilon_{u_2}^2 \frac{k_0^4}{r_0^{\,2}} |F(qR)|^2 \int_0^{2\pi}\int_0^{\pi}\int_0^{2\pi} \left| \sum_{xyz} G(\theta;\psi,\xi,\zeta)_{xyz} \right|^2 d\zeta \sin\xi d\xi d\psi \quad (7)$$

Here the modulus squared of *G(Θ;ψ,ξ,ς)* is isotropically averaged over all Euler angles. This equation can be solved for all polarization combinations. Of particular relevance in this work are I_{SSS} and I_{PSS}. If we work out the integral over all Euler angles for *G(Θ;ψ,ξ,ς)* for T_d symmetry, which has a single independent tensor element *xyz=xzy=yzx=yxz=zxy=zyx*,[3,4] we obtain the following expressions for I_{SSS} and I_{PSS}:

$$I_{PSS} = I_{u_{0,p},u_{1,s},u_{2,s}}(r_0) \propto \int_0^{2\pi}\int_0^{\pi}\int_0^{2\pi} \left| \sum_{xyz} G(\theta;\psi,\xi,\zeta)_{xyz} \right|^2 d\zeta \sin\xi d\xi d\psi \propto \frac{\beta_{xyz}^2}{420}(91\cos^2(\theta)+96\sin^2(\theta))$$

(8)

$$I_{SSS} = I_{u_{0,s},u_{1,s},u_{2,s}}(r_0) \propto \int_0^{2\pi}\int_0^{\pi}\int_0^{2\pi} \left| \sum_{xyz} G(\theta;\psi,\xi,\zeta)_{xyz} \right|^2 d\zeta \sin\xi d\xi d\psi \propto \frac{12\beta_{xyz}^2}{35} \quad (9)$$

At 90° we find that the relative contribution of I_{SSS}/I_{PSS} equals 2/3 as expected for T_d symmetry. Notice that I_{SSS} is independent of scattering angle *Θ*. There is a slight angle dependence for I_{PSS}, so that the depolarization becomes larger towards scattering angles lower or higher than 90°. This angle dependence must be taken into account as well, so we arrive at the following equations for T_d symmetry:

$$I_{PSS} + I_{SSS} \propto \text{scale.}|F(qR)|^2 \left(\frac{12}{35} + \frac{1}{420}(91\cos^2(\theta)+96\sin^2(\theta))\right) \quad (10)$$

$$I_{SSS} \propto \frac{12}{35}.\text{scale.}|F(qR)|^2 \quad (11)$$

The scaling factor is used as a measure of concentration as explained in the manuscript. In this work we chose to fit the $I_{SSS}+I_{PSS}$ curves since the signal level is higher.

The depolarization ((i.e. I_{SSS}/I_{PSS} with I_{xxx} the SH light intensity with *xxx* polarization combination) was measured here by comparing AR-SHS curves with and without polarizing sheet present (see Figure 2

article). The intensity with polarizer present corresponds to T.I_{SSS}, where T stands for the transmission of the polarizing sheet at the second harmonic wavelength (measured with Perkin-Elmer UV-VIS spectrometer). Without polarizer present the intensity corresponds to $I_{SSS}+I_{PSS}$. Typical curves are shown in Supplementary Figure 4a. The depolarization was found by integrating the signal over all angles with and without an analyzer present in order to find the relative contribution of *SSS* and *PSS* polarization combinations, taking into account the transmission of the polarizing sheet. Taking into account the angle dependence of the depolarization by integrating over all angles used in the data-analysis we expect a value of 0.64 for T_d symmetry. In Supplementary Figure 4bthe normalized data are shown with and without polarizing sheet.

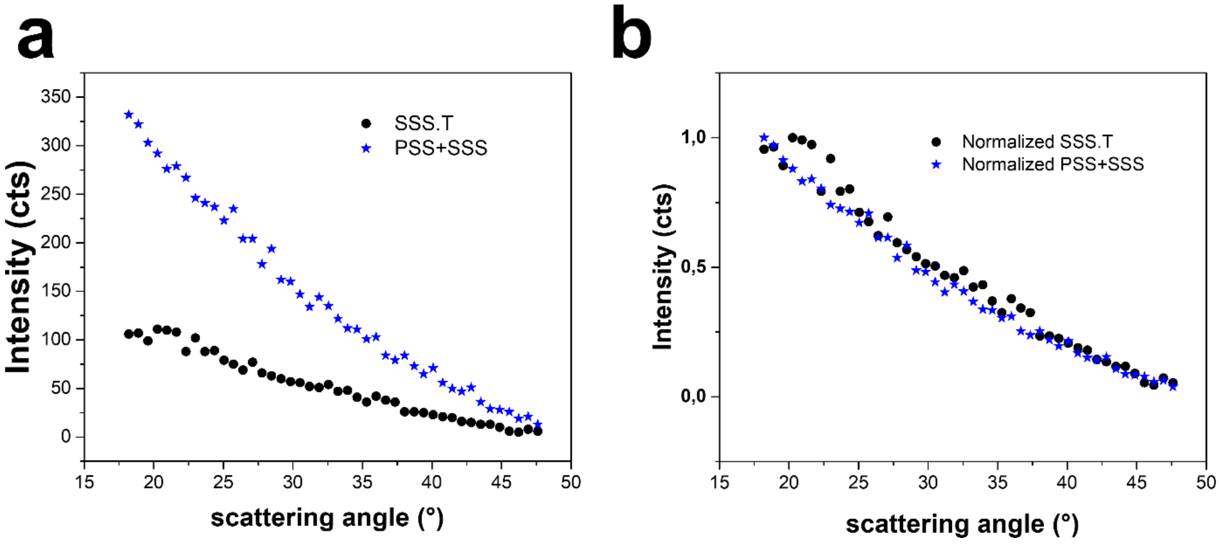

Supplementary Figure 4. AR-SHS patterns during growth of ZIF-8. a) AR-SHS during growth at 12 minutes, with and without polarizing sheet present. SSS and PSS represent the measured polarization combinations, T stands for the transmission of the polarization sheet. b) Same data normalized to the maximum value.

In Figure SI.6 some typical fits of the $I_{SSS}+I_{PSS}$ patterns to equation:

$$I_{PSS}+I_{SSS} \propto \text{scale.}\left|F(qR)\right|^2 \left(\frac{12}{35}+\frac{1}{420}(91\cos^2(\theta)+96\sin^2(\theta)\right) \quad (12)$$

are shown. These were obtained by nonlinear least squares regression in OriginPro. The fit values are: *R*=68±33 nm; 180±5 nm; 233±10nm; 310±3 nm; 371±2nm; 417±3 nm/ *scale*= 1.02 ± 3.23 E-11; 7.08 ± 1.06 E-14; 2.01 ± 0.06 E-14; 2.33 ± 0.01 E-14; 2.50 ± 0.07 E-14; 2.88 ± 0.08 E-14 for 5; 6.5; 7.5; 10; 12.5; 15 minutes respectively. Note that the curves at 5 and 6.5 minutes are averages over 30 seconds (10 measurements).

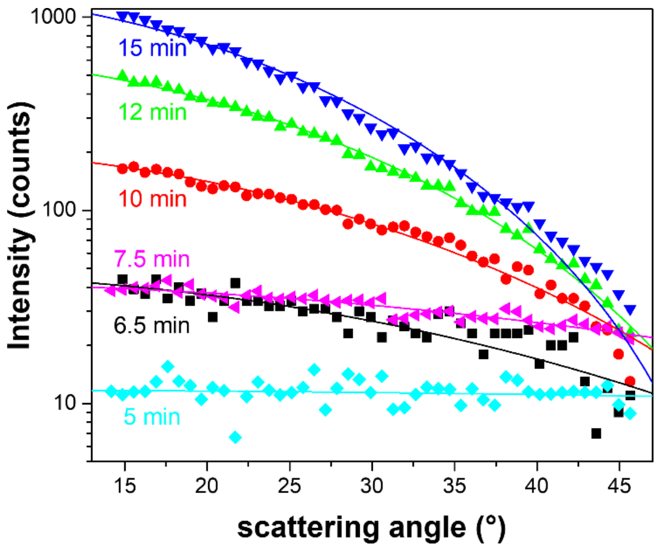

Supplementary Figure 5. Fits to the nonlinear Rayleigh-Gans-Debye model during growth of ZIF-8. The solid lines represent the best fit to the data at different growth times obtained by nonlinear regression, assuming a monodisperse distribution of spheres (Equation 12).

Supplementary Note 4. Second harmonic scattering during crystal growth of ZIF-8

The SHS data obtained during crystallization of ZIF-8 are shown in Supplementary Figure 6. For experimental details see methods. The original data are represented as red dots. Since the input power was changed during the experiment a correction has been made assuming a quadratic dependence of the SHS intensity on the fundamental light intensity. This correction results in smooth corrected curves which implies that higher order effects have no significant impact for the intensities used here. The time resolution was 3 seconds. Remark that for the first 2 minutes the data were averaged over 10 measurements.

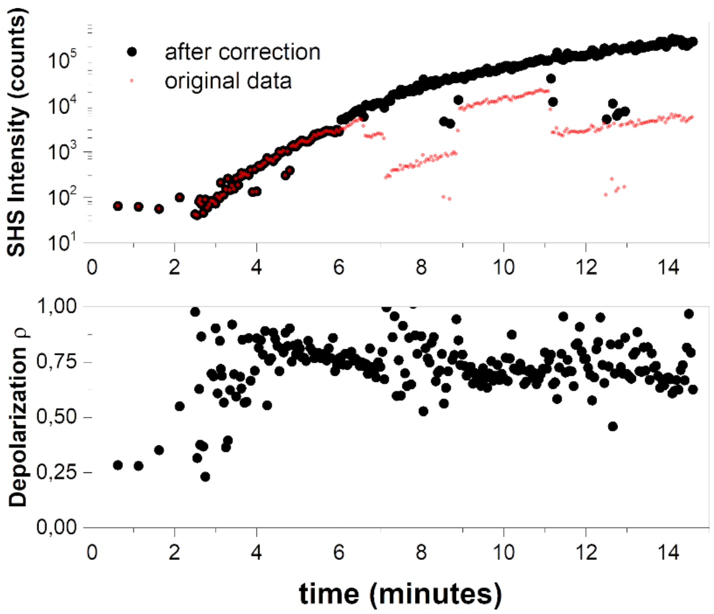

Supplementary Figure 6. SHS measurement during crystal growth of ZIF-8, in transmission mode. Since the input power was changed during the experiment a correction has been made assuming a quadratic dependence of the SHS intensity on the fundamental light intensity. Simultaneous measurement of different polarization combinations allowed extraction of the depolarization.

Supplementary Note 5. Analysis towards shape or size distribution

For analysis towards shape or size distribution models developed for linear scattering methods can be applied. A package with a wide range of models is available through the NIST center for neutron research (https://www.ncnr.nist.gov/programs/sans/data/data_anal.html).

In Supplementary Figure 6 the AR-SHS data at 15 minutes are fitted with form factor functions for a spherical and cylindrical shape model.[5] The fit parameters for the spherical model are scale=4.93±0.08 E-15; 7.75±0.02E-15/ radius=364±3 nm; 396±3 nm for 12 and 15 minutes. For the cylindrical model we obtain length= 434±287 nm; 568±16 nm/ radius=143±292 nm; 255±19 nm at 12 and 15 minutes. For the Gaussian spheres model (GaussSpheres, NIST) we obtain scale=9.38±0.03 E-14; 1.81±0.03 E-13/ radius=357±0.11 nm; 343±22 nm/ sigma σ=22±17 nm; 77±15 nm for 12 and 15 minutes.

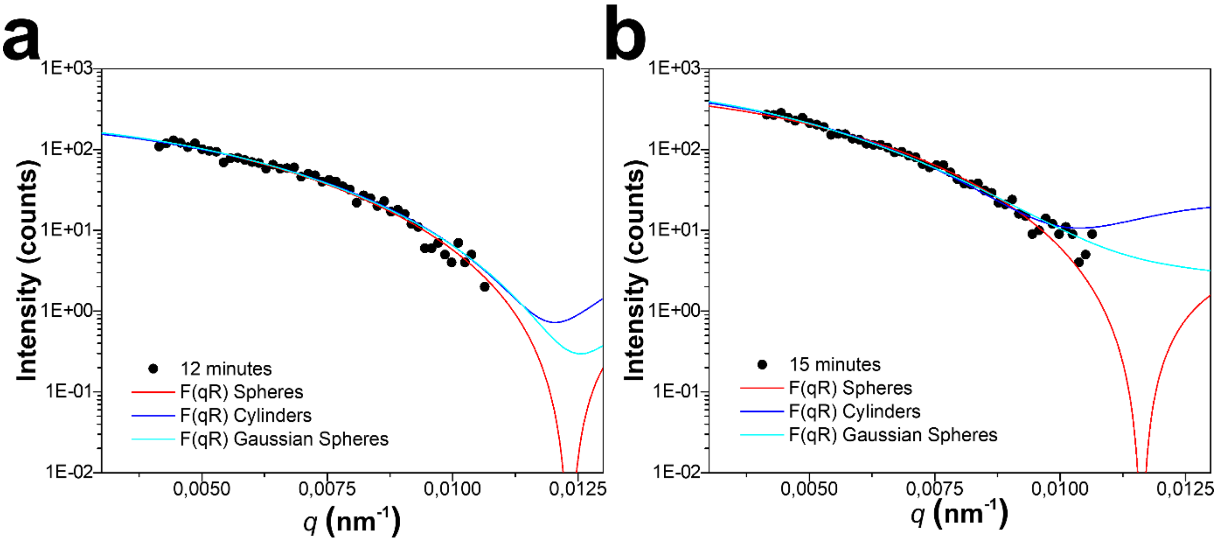

Supplementary Figure 7. Analysis of AR-SHS data during crystal growth of ZIF-8 towards form factor. Fit to form factor function for spherical and cylindrical particles, and for a Gaussian distribution of spheres at a) 12 and b) 15 minutes.

Supplementary Note 6. Calibration towards concentration

This requires measurement of the magnitude of the nonlinear susceptibility $\chi^{(2)}$ for the material under study, e.g. by measurement of second harmonic generation against a known standard such as quartz, as some of the authors showed in earlier work.[6] Once the nonlinear susceptibility $\chi^{(2)}$ of the material is known the detection efficiency of the setup must be calibrated. This can be achieved by subsequent calibration of the detected intensity for a standard solvent with known first hyperpolarizabilty β. The response of the system can then be described by G.N. $\beta^2.I^2$, with G a constant accounting for the efficiency of the setup, N the concentration of solvent molecules, β the first hyperpolarizabilty and I the fundamental intensity. G is determined in this way. If G, β and I are known the concentration can be found for other compounds as well. By applying the same procedure to Eq.1 from the article the concentration N can in principle be found. In practice such a procedure is tedious, since it ultimately relies on a correct determination of the nonlinear susceptibility $\chi^{(2)}$ which is time-consuming. Moreover standard methods such as the Kurtz powder method are semi-quantitative and prone to errors.

Supplementary Note 7. Kinetic Avrami model

In Figure SI-8 we plotted the number of particles (N) multiplied with the cube of the particle radius (R^3), both parameters extracted from the model presented above, as a function of time. Assuming a collection of spherical particles with identical structure (as implied by the depolarization), NR^3 is proportional to the total particle mass of crystalline ZIF-8 and hence a measure of the extent of crystallization α. A fit to the well-known Avrami model with nonlinear least squares fitting in Originpro is shown as a blue line.[7] Fit parameters are k (rate constant)=4.19E-8±8.54 E-5; n(Avrami exponent)= 2.70±0.09; Maximum (α→1)=0.034±68.57. The large errors on the fitted values of the rate constant and the maximum can be due to i) the fact that only the beginning of the crystallization curve is available and ii) because the Avrami model fudges the mechanism as a one-step process, which is not the case here. Fitting exclusively to the data obtained after 8 minutes resulted in k (rate constant)=3.48 E-7 ± 8.00 E-5; n(Avrami exponent)= 2.73±0.10; Maximum (α→1)=0.037± 0.86. In general, and here even more so, interpretation of the Avrami model in terms of mechanism is difficult, but nonetheless a change in mechanism before and after 8 minutes can be inferred from these data. Before 8 minutes the particle mass seems to remain rather constant, while at the same time the particle radius increases. After 8 minutes the mass increases exponentially.

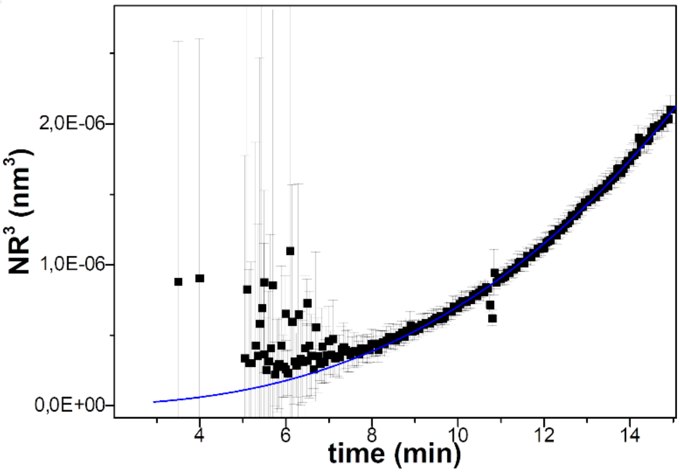

Supplementary Figure 8. Kinetic analysis of the crystallization of ZIF-8. From the values obtained for the radius (R) and the number of particles (N) by nonlinear regression towards the nonlinear Rayleigh-Gans-Debye model for monodisperse spheres during the crystal growth of ZIF-8, NR^3 was calculated as a measure of the total mass of ZIF-8 in solution. The values for NR^3 at different times during the synthesis were fitted to the kinetic Avrami model (blue line). The standard errors for the derived parameters are estimated by the OriginPro fitting software according to the Error Propagation formula.

Supplementary Note 8. Repeated dAR-SHS measurements during crystal growth of ZIF-8

In Supplementary Figure 9 the results of 2 repeated dAR-SHS measurements are depicted for the same synthesis procedure, giving similar results.

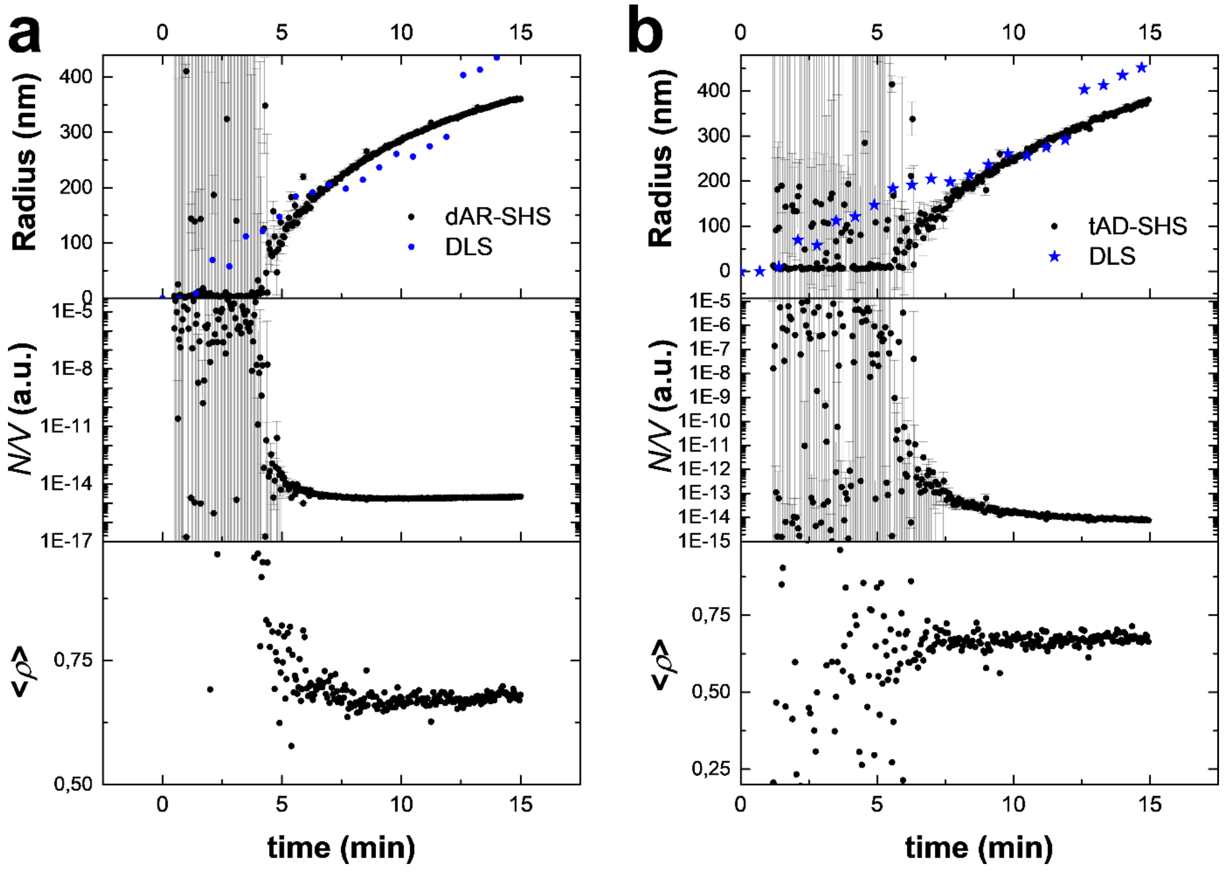

Supplementary Figure 9. Repeated dAR-SHS measurements. Panels a) and b) represent 2 repeated dAR-SHS measurements during crystal growth of ZIF-8 starting from the same stock solutions. Radius, number of particles (N) were extracted by nonlinear regression towards the nonlinear Rayleigh-Gans-Debye model for monodisperse spheres. The standard errors for the derived parameters are estimated by the OriginPro fitting software according to the Error Propagation formula. The average depolarization <*ρ*> approaches 0.64, as expected for the symmetry group of ZIF-8 (T_d).

1. Wang, H., Yan, E. C. Y., Borguet, E. & Eisenthal, K. B. Second harmonic generation from the surface of centrosymmetric particles in bulk solution. *Chem. Phys. Lett.* **259,** 15–20 (1996).

2. Yang, N., Angerer, W. E. & Yodh, a G. Angle-resolved second-harmonic light scattering from colloidal particles. *Phys. Rev. Lett.* **87,** 103902 (2001).

3. Boyd, R. W. *Nonlinear optics*. (Academic press, 2003).

4. Verbiest, T., Clays, K. & Rodriguez, V. *Second-order Nonlinear Optical Characterization Techniques: An Introduction*. (CRC Press, 2009).

5. Rice, S. A. Small angle scattering of X-rays. A. Guinier and G. Fournet. Translated by C. B. Wilson and with a bibliographical appendix by K. L. Yudowitch. Wiley, New York, 1955. 268 pp. $7.50. *J. Polym. Sci.* **19,** 594–594 (1956).

6. Van Cleuvenbergen, S. *et al.* ZIF-8 as Nonlinear Optical Material: Influence of Structure and Synthesis. *Chem. Mater.* **28,** 3203–3209 (2016).

7. Avrami, M. Granulation, Phase Change, and Microstructure Kinetics of Phase Change. III. *J. Chem. Phys.* **9,** 177 (1941).
